# The relationship between quality of work life and turnover intention of primary health care nurses in Saudi Arabia

**DOI:** 10.1186/1472-6963-12-314

**Published:** 2012-09-12

**Authors:** Mohammed J Almalki, Gerry FitzGerald, Michele Clark

**Affiliations:** 1 Faculty of Public Health and Tropical Medicine, Jazan University, Jazan, Saudi Arabia; 2 School of Public Health and Institute of Health and Biomedical Innovation (IHBI), Queensland University of Technology, Kelvin Grove, QLD, Australia

**Keywords:** Nurse, Nursing workforce, Primary health care, Quality of work life (QWL), Saudi Arabia, Turnover intention

## Abstract

**Background:**

Quality of work life (QWL) has been found to influence the commitment of health professionals, including nurses. However, reliable information on QWL and turnover intention of primary health care (PHC) nurses is limited. The aim of this study was to examine the relationship between QWL and turnover intention of PHC nurses in Saudi Arabia.

**Methods:**

A cross-sectional survey was used in this study. Data were collected using Brooks’ survey of Quality of Nursing Work Life, the Anticipated Turnover Scale and demographic data questions. A total of 508 PHC nurses in the Jazan Region, Saudi Arabia, completed the questionnaire (RR = 87%). Descriptive statistics, *t*-test, ANOVA, General Linear Model (GLM) univariate analysis, standard multiple regression, and hierarchical multiple regression were applied for analysis using SPSS v17 for Windows.

**Results:**

Findings suggested that the respondents were dissatisfied with their work life, with almost 40% indicating a turnover intention from their current PHC centres. Turnover intention was significantly related to QWL. Using standard multiple regression, 26% of the variance in turnover intention was explained by QWL, *p* < 0.001, with R^2^ = .263. Further analysis using hierarchical multiple regression found that the total variance explained by the model as a whole (demographics and QWL) was 32.1%, *p* < 0.001. QWL explained an additional 19% of the variance in turnover intention, after controlling for demographic variables.

**Conclusions:**

Creating and maintaining a healthy work life for PHC nurses is very important to improve their work satisfaction, reduce turnover, enhance productivity and improve nursing care outcomes.

## Background

Nurse turnover has been a major challenge for many health care organisations. Turnover of qualified nurses has consequences for health organisations as well as the profession as a whole. Nurse turnover can have a negative impact on the capacity to meet patient needs and provide quality care [1]. In addition, the loss of nurses leads to inadequate staffing, which in turn, may decrease morale and create more stress on the ‘stayers’ due to increased workloads [2-4]. This can lead to critical changes in the behaviour of nurses towards their jobs resulting in low work satisfaction, low productivity, and finally, leaving the organisation. Additionally, without adequate and experienced staff, error rates may increase and patient satisfaction may decrease [5]. Nurse turnover is also costly for healthcare organisations and it “consumes resources that could be directed at core business activities, such as quality improvement programs, and staff development or nurse retention activities” [6] (p. 562). Previous research has argued the importance of quality of work life (QWL) to the commitment of health professionals, including nurses [7,8]. Brooks [9] defined QWL as “the degree to which registered nurses are able to satisfy important personal needs through their experiences in their work organisation while achieving the organisation’s goals” (p. 9). Assessing QWL allows organisations to understand how work environments and home life challenges affect the nurses’ work experience, work satisfaction and organisational commitments [8,10]. According to Lees and Kearns [11], high QWL is essential for organisations to attract new employees and retain their workforces. However, research studies on QWL and turnover intention of primary health care (PHC) nurses are limited.

A number of studies have explored QWL among nurses; however, the majority comes from hospital-based research in western countries. To date, no such studies focus specifically on PHC nurses. Nor is there any published research on the relationship between QWL and turnover intention among this category of nurses. There is a real need to conduct further studies of QWL in different health settings, including PHC facilities. This need is increased in Saudi Arabia, which has a chronic shortage of Saudi health care professionals, especially nurses, which is accompanied by a high level of turnover [12,13].

Findings from a few studies conducted in the major cities of Saudi Arabia have indicated that nurses, particularly PHC nurses, are dissatisfied with their work [14,15]. According to Al Juhani and Kishk [14], in a survey study conducted in the Al-Madinah region to assess the level of work satisfaction among PHC professionals, 52.4% of staff nurses were highly dissatisfied. This dissatisfaction among PHC nurses can have a negative impact on their performance and in turn affect the quality of healthcare outcomes. Furthermore, it can result in a behavioural intention to leave their work, which they may do ultimately.

Previous studies of PHC nurses in Saudi Arabia focused on job satisfaction only, omitting other important factors such as work life/home life, work design, work context and other external factors, which form the QWL approach [16]. Consequently, there is a need to conduct a research study to explore and assess QWL and related factors among PHC nurses in the Saudi health system. The findings may assist in developing strategies to attract and retain more nurses to PHC organisations, particularly during this era of transition into PHC. Otherwise, in light of competition to attract qualified nurses, the PHC centres in Saudi Arabia may lose skilled nurses who may prefer to work for other systems and organisations, either nationally or internationally, that provide appropriate working environments. According to Alamri et al. [17], a number of expatriate nurses leave Saudi Arabia as soon as they have obtained sufficient experience to work in developed countries.

This study is part of a larger study aiming to improve the retention of PHC nurses in the Jazan region, Saudi Arabia, through exploring and assessing their QWL and turnover intention. The purpose of the present paper, therefore, was to examine the relationship between QWL and turnover intention of PHC nurses in the Jazan region, Saudi Arabia. The main questions of this study were as follows: (a) are there significant relationships between turnover intention and the selected demographic variables of PHC nurses; and (b) are the QWL dimensions (i.e. work life/home life, work design, work context and work world) useful in predicting turnover intention.

### Primary healthcare in Saudi Arabia

Saudi Arabia is one of the leading countries that has adopted and implemented the PHC approach in the Middle East [18]. In accordance with the Alma-Ata declaration at the WHO General Assembly in 1978, a ministerial decree was issued in Saudi Arabia to integrate the existing services such as former health offices, maternal and child health centres and small dispensaries into single unites named PHC centres [19]. PHC centres supply primary healthcare services, both preventive and curative, to the public. Using a structured referral system, cases that require advanced care are referred to the second level of care (public hospitals). Cases that need more complex levels of care are transferred to the third level of health care (referral hospitals) [19]. The top-to-down relationship between healthcare organisations at various levels is not clearly organised. For example, there are no communication channels or planned regulations for sending patients back to PHC services from specialist or secondary care sectors [20].

The Ministry of Health in Saudi Arabia (MOH) found that approximately 82% of client visits to its facilities during 2009 were to PHC centres, comprising more than 54 million PHC clients [21]. Taking such data into account, the MOH has continued to develop healthcare services in general and PHC in particular by initiating many contemporary projects. The best example for such development is the project of the Custodian of the Two Holy Mosques that aims to establish 2000 advanced PHC centres and to develop the existing ones in terms of buildings, capital resources, workforce and services provided [19].

Due to the importance of human resources in providing quality PHC services, it is integral for PHC leaders to assess their QWL and to understand their organisational and career intentions. Such procedures may assure the continuous and improvement of the health services being provided.

### A theoretical framework

A theoretical framework was developed to guide this study. It incorporates the four-dimension model of Quality of Nursing Work Life (QNWL) (work life/home life, work design, work context and work world) [16], and a set of demographic variables that were reported in the literature as variables commonly correlated with nurses’ satisfaction and turnover intention. The ‘work life/home life’ dimension refers to “the interface between the life experiences of nurses in their place of work and in the home”, while ‘work design’ is “the composition of nursing work, and describes the actual work nurses do” [16]. The third dimension of ‘work context’ encompasses “the practice settings in which nurses work and explores the impact of the work environment on both nurse and patient systems”, while ‘work world’ takes account of “the effects of broad societal influences and changes on the practice of nursing” [16].

Demographic variables incorporated into the QWL and Turnover Intention Model include gender, age, marital status, dependent children, dependent adults, nationality, ethnicity, level of education, nursing tenure, organisational tenure, positional tenure, location of the PHC, and payment per month. Demographic variables are frequently used in nursing research as predictors of turnover and turnover intention [22]. According to Bluedom [23], demographic variables display independent effects: thus, they could not be omitted as contributors to turnover.

**Figure 1:**
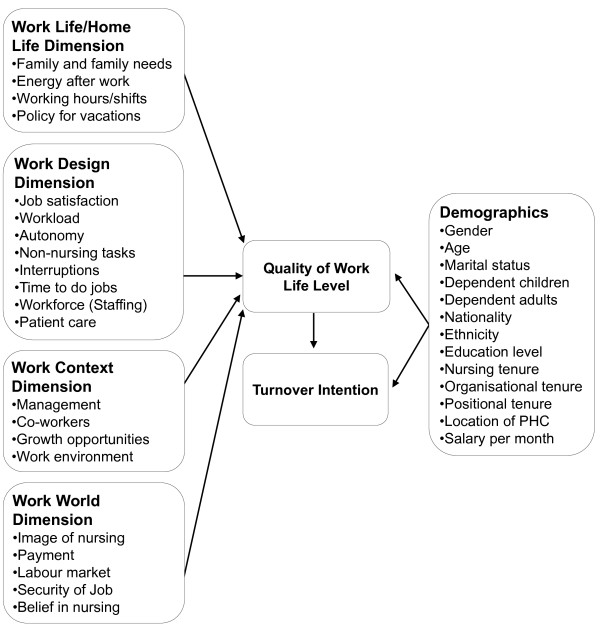
QWL and turnover intention framework.

The proposed model assumes a correlation among work life related factors, employees’ demographic characteristics, QWL level and turnover intention. QWL is affected by work life factors as presented by Brooks and Anderson [16] as well as a number of demographic factors, leading either to a high or low level of QWL, which can result in critical changes in the behavioural intention of the nursing employee. This intention is translated into the behaviour action of staying with the current organisation or leaving. The correlation between the level of QWL and the employees’ behaviour action, however, can be further affected by the selected employees’ demographic factors. For example, when QWL is low, it is expected that this would lead to turnover intention, and finally, to actual turnover. In some cases, besides the QWL level, the employees’ demographic factors may have a significant impact on the final decision of turnover (see Figure 1). 

## Methods

### Design and sample

A cross-sectional survey was used in this study. All PHC nurses in the Jazan region, Saudi Arabia, were eligible for inclusion in the sample. There were 134 PHC centres employing approximately 585 Saudi and non-Saudi nurses. A total of 508 PHC nurses returned a usable questionnaire, yielding an 87% response rate.

### Instruments

In addition to the demographic questions, two instruments where used in this study. These include the Anticipated Turnover Scale (ATS) and the Brooks’ survey of QNWL. The ATS survey was developed by Hinshaw and Atwood in 1978 to study turnover intention among nurses [24]. The ATS is a 12-item self-administered instrument with a 7-point Likert scale ranging from ‘agree strongly’ to ‘disagree strongly’ [24]. The instrument’s items were related to an employee’s anticipated length of time to leave and certainty of leaving the job. The total score was obtained by calculating the sum of all items in the scale divided by the number of items in the scale. Higher scores reflect greater intent to leave the present position or job. Responses with means over 3.5 were considered as an indication for turnover intention [25]. According to Hinshaw and Atwood [24], the construct validity for the ATS was estimated using principal component factor analysis. Findings identified two factors which accounted for 55% of the variance. The internal consistency reliability estimated with Cronbach’s α was .84 [24]. The ATS has been used frequently to measure turnover intention among nursing workforces in various healthcare settings [25-32]. Barlow and Zangoro [33] conducted a meta-analysis study aimed at determining the consistency of reliability estimates and evidence of construct validity of the ATS scores across nursing studies in the USA. The overall mean weighted effect size of reliability from 12 studies was .89, indicating excellent reliability and construct validity. In the present study, Cronbach’s alpha for ATS is (.90).

The QNWL survey was developed by Brooks [9] to measure QWL among Registered Nurses. It consists of 42 items related to four dimensions: (a) work life/home life, (b) work design, (c) work context and (d) work world. The instrument asks respondent nurses how much they agree or disagree with each item on a 6-point scale ranging from 1 ‘strongly disagree’ to 6 ‘strongly agree’. The test-retest reliability was determined in a traditional 14-day manner with Pearson’s *r* = .90 (*n* = 53), where 1.00 indicated ‘perfect’ reliability. In terms of construct validity, the total calculated for the 42-item survey using Cronbach’s α is .89 (*n* = 265) where 1 indicates ‘perfect’ validity [9]. In this study, Cronbach’s alpha for Brooks’ scale is (.89). Brooks’ survey of QNWL has been used by other published works in the USA and Iran [10,16,34,35], with increasing interest globally [36]. The instruments used were contextualized to the local context and the multicultural nursing workforce in Saudi Arabia. In addition to the original English format, the questionnaire used was translated into Arabic using a translating and back translated technique and a committee approach [37]. Two bilingual researchers blindly translated the instruments: one from English to Arabic, and the other back-translated the questionnaire [37]. A panel of three bilingual experts in health research and in health management reviewed the questionnaire and assured its validity. Two pilot studies were conducted to ensure the clarity and appropriateness of the questionnaire [20].

### Data collection and analysis

Following permission from the MOH in Saudi Arabia to conduct the study, ethics approval from Queensland University of Technology was obtained (no.0800000406). The survey was sent to PHC nurses through the Department of PHC in Jazan. Each nurse was provided with a survey package including a cover letter, questionnaire, and an individual envelope. The cover letter explained the research, provided contact details of the researchers, and outlined the steps taken to maintain confidentiality. No names or other identifiable information of respondents were required. Each respondent in every PHC centre was requested through the information letter to seal the completed survey separately within the provided individual envelope. Then, all nurses in the same workplace were instructed to seal their surveys together in another labelled large envelope (provided) and return them to the Department of PHC through the internal mail service. Participants were informed of the voluntary nature of participation. The returned completed questionnaires indicated consent to participate. More details on the data collection procedure are reported in another paper published elsewhere [38]. Descriptive statistics, *t*-test, ANOVA, General Linear Model (GLM) univariate analysis, standard multiple regression, and hierarchical multiple regression were applied for analysis using SPSS v17 for Windows.

## Results

The majority of nurses were Arab (73.8%), Saudi (72.2%), females (67.3%) and aged between 20 and 29 years (44.1%). The majority of respondents declared that they were married (73.8%), and had children (61%) and/or dependent adults (54.9%). Approximately half of the sample (47.4%) had a Diploma, 33.7% had an Institute Certificate, 12.8% had an Associate Degree, and 5.3% had a Bachelor Degree or higher. About 46% of the sample received a monthly salary of 5,000 to 10,000 Saudi Riyals (SR) (1US$ = SR 3.75) (46.3%). Among the respondents, 62% stated that they cover two departments or more during their duties. The mean work experience as an RN was 11.3 years, with about 6.6 years in the current PHC organisation and 6.1 years in the current position.

The total possible score for Brooks’ scale can range from 42–252. A low total scale score indicates a low overall QWL, while a high total score indicates a high QWL. Respondents had a range score of 45–218 (*M* = 139.45), which is lower than the average score on Brooks’ scale, indicating that the respondents were dissatisfied with their work life [39]. This part of the findings will be reported and discussed elsewhere [38]. In terms of turnover intention, findings suggested that about 40% of the respondents indicated that they intended to leave their current PHC centre.

### Demographic variables and turnover intention

An independent samples *t*-test and an ANOVA were conducted to examine if there is any relationship between the turnover intention and the demographic variables. Significant associations were found between turnover intention and demographic variables of gender, age, marital status, dependent children, education level, nursing tenure, organisational tenure, positional tenure, and payment per month. The eta squared test for these demographics indicates small to medium effect size of the variation in turnover intention scores. The associations between turnover intention and demographic variables of dependent adults, nationality, ethnicity, and location of PHC were not significant. Results of *t*-test and ANOVA procedures are presented in Table 1. The demographics were also examined as a set using the GLM univariate. The results found that only four demographic variables had strong relationships with turnover intention, *p <* 0.05. These were gender, dependent adults, positional tenure and payment per month. However, the model as a whole is still significant. Altogether, the demographic variables explain 11.1% (Adjusted R^2^ = .111) of the variability in turnover intention, *F*(4,444) = 14.976, *p* < 0.001. Table 2 presents the parameter estimates for the significant demographic variables on the turnover intention scores.

**Table 1:** **Turnover intention by demographic variables using *t *-test and ANOVA **

**Variable**	**Mean**	**SD**	**t/F-value**	**P-value**
**Gender**				
Male	43.99	13.61	3.48	0.001
Female	39.71	11.62		
**Age**				
20-29 years	43.20	11.87	7.24	<0.001
30-39 years	41.79	13.48		
40-49 years	35.93	11.58		
50-59 years	38.36	9.46		
**Marital status**				
Never married	43.37	12.89	2.98	0.052
Married	40.48	12.29		
Divorced/Widowed	37.42	12.21		
**Dependent children**				
Yes	39.97	12.56	−2.59	0.010
No	42.89	12.12		
**Education level**				
Institute	38.87	13.66	3.12	0.026
Diploma	41.86	11.73		
Associate	43.66	12.69		
Bachelor or higher	41.96	8.75		
**Nursing tenure**				
≤ 4 years	44.37	130.05	9.49	<0.001
5-9 years	41.93	10.76		
≥ 10 years	38.82	12.51		
**Organisational tenure**				
≤ 4 years	43.52	12.21	16.50	<0.001
5-9 years	41.06	11.50		
≥ 10 years	36.28	12.31		
**Positional tenure**				
≤ 4 years	43.84	12.45	20.64	<0.001
5-9 years	39.92	10.92		
≥ 10 years	35.62	11.85		
**Payment per month**				
< SR 5,000	42.45	10.53	9.60	<0.001
SR 5,000-10,000	42.98	12.80		
> SR 10,000	37.25	12.82		

**Table 2:** Parameter estimates of the demographic variables on the turnover intention using the GLM univariate analysis

**Parameter**	**Estimates**	**Std. Error**	**t**	**Sig.**	**95% Confidence Interval**	
					**Lower Bound**	**Upper Bound**
Intercept	46.069	1.546	29.804	<0.001	43.031	49.107
Gender						
1 = Male	4.371	1.286	3.399	0.001	1.844	6.898
2 = Female	0^a^	.	.	.	.	.
Dependent adults						
1 = Yes	2.238	1.162	1.925	0.055	-.047	4.522
2 = No	0^a^	.	.	.	.	.
Positional tenure	-.350	.092	−3.792	<0.001	-.532	-.169
Payment per month	-.644	.197	−3.261	0.001	−1.032	-.256

a. This parameter is set to zero because it is redundant.

*Note.* Dependent variable: Turnover Intention. Only significant demographic variables and intercepts were presented in this table. R Squared = .133 (Adjusted R Squared = .098).

### QWL and turnover intention results

A standard multiple regression was performed between turnover intention as the dependent variable and the four dimensions of QWL (work life/home life, work design, work context, and work world). Altogether, 26% of the turnover intention among the PHC nurses was explained by knowing the scores for the four dimensions of QWL. R for regression was significantly different from zero, *F*(4,491), 43.71, *p* < 0.001, with R^2^ = .263 (see Table 3).

**Table 3:** Model summary for standard multiple regression of the QWL dimensions on the turnover intention scores

**Model**	**R**	**R**^**2**^	**Adjusted R**^**2**^	**Std. Error of the Estimate**
1	.512^a^	.263	.257	10.625

a. Independent Variables: (Constant), Work life/Home life, Work Design, Work Context, Work World.

*Note.* Dependent Variable: Turnover intention.

The largest beta value in this case was -.387, which is for work context, followed by work design (−.112). This means that the work context variable makes the strongest unique contribution to explaining turnover intention, when the variance explained by all other variables in the model was controlled. The other significant variable was work design *p* < 0.05. Its beta value (−.112) was lower than the work context value, indicating that it made less of a contribution. Although the bivariate correlations between turnover intention and each of the work life/home life and work world dimensions were statistically different from zero (see Table 4), they did not contribute significantly to the explanation of turnover intention. Apparently, the relationship between turnover intention and each of the work life/home life and work world dimensions are mediated by the relationships between the other independent variables (work design and work context) and turnover intention. Table 5 displays the unstandardised coefficients (B), standard error, standardised coefficients (β), *t* value and the significance of model for the standard multiple regression.

**Table 4:** Correlation between QWL variables and turnover intention

**Variables**	Turnover Intention*	**Work life/home life**	**Work Design**	**Work Context**
Work life/home life	-.245**			
Work Design	-.408**	.446**		
Work Context	-.497**	.424**	.667**	
Work World	-.291**	.309**	.444**	.418**

* Dependent variable. ** Correlation is significant at the 0.01 level.

**Table 5:** Summary of coefficients for the standard multiple regression of the QWL dimensions on the turnover intention scores

	**Model**	**Unstandardized Coefficients**		**Standardized Coefficients**	**t**	**Sig.**
	**Variable**	**B**	**Std. Error (SE)**	**Beta (β)**		
1	(Constant)	79.878	3.210		24.888	<0.001
	Work life/home life	-.018	.107	-.007	-.169	0.866
	Work design	-.208	.102	-.112*	−2.041	0.042
	Work context	-.394	0.055	-.387**	−7.209	<0.001
	Work world	-.270	.155	-.077	−1.740	0.082

*Note.* Dependent variable is turnover intention. * *p* < 0.05. ** *p* < 0.001.

The hierarchical multiple regression analysis was used to examine if the QWL dimensions are still useful in predicting turnover intention after controlling for the possible effect of the demographic variables. The demographics were entered at Step 1, explaining 13.2% of the variance in turnover intention. After entry of the four dimensions of QWL (work life/home life, work design, work context and work world) at Step 2, the total variance explained by the model as a whole was 32.1%, *F*(17.433) = 12.04, *p* < 0.001. The four QWL dimensions explained an additional 19% of the variance in turnover intention, after controlling for demographic variables, R squared change = .19, *F* change (4, 433) = 30.190, *p* < 0.001. In the final model, only four variables where statistically significant: work context recording a highest beta (β = −.37, *p* < 0.001), followed by positional tenure (β = −.30, *p* < 0.05), payment per month (β = −.23, *p* < 0.05), and finally, gender (β = −.11, *p* < 0.05). Table 6 displays the unstandardised coefficients (B), standard error, standardised coefficients (β), *t* value and the significance of the model for hierarchical multiple regression.

**Table 6:** Summary of coefficients for hierarchical multiple regression of the QWL dimensions on the turnover intention scores after controlling for demographic variables

**Model**		**Unstandardized Coefficients**		**Standardized Coefficients**	**t**	**Sig.**
**Variable**		**B**	**Std. Error (SE)**	**Beta (β)**		
Step 1	(Constant)	59.524	6.135		9.702	<0.001
	Gender	−4.405	1.394	-.168*	−3.159	0.002
	Positional tenure	-.543	.229	-.301*	−2.368	0.018
	Payment per month	−1.310	.393	-.361**	−3.336	0.001
Step 2	(Constant)	87.826	6.169		14.235	<0.001
	Gender	−2.907	1.253	-.111*	−2.319	0.021
	Positional tenure	-.532	.204	-.295*	−2.602	0.010
	Payment per month	-.840	.353	-.231*	−2.381	0.018
	Work life/home life	-.047	.127	-.019	-.366	0.715
	Work design	-.128	.109	-.069	−1.174	0.241
	Work context	-.380	0.058	-.374**	−6.507	<0.001
	Work world	-.225	.162	-.064	−1.386	0.166

*Note.* R^2^ = .132 (Adjusted R^2^ = .106) for Step 1, R^2^ = .321 (Adjusted R^2^ = .294) for Step 2 (*p* < 0.001). Only significant coefficients and QWL dimensions are presented in this table. * *p* < 0.05. ** *p* < 0.001.

## Discussion

The findings of the present study indicated that the respondents were dissatisfied with their work life. These findings are consistent with findings of a number of previous studies where nurses were not satisfied with their work life [10,34,40]. Successful QWL strategies in healthcare settings can improve employees’ morale and organisational effectiveness [41]. Additionally, QWL can improve the quality of care provided as well as recruitment and retention of the nursing workforce [36,42].

Using the ATS, about forty percent (40.4%, *n* = 205) of the respondent nurses indicated that they intended to leave their current employment. This finding supports the notion that turnover and turnover intention are high among nurses in general [43-46], and among nurses working in Saudi Arabia [47-49]. Saeed [49] conducted a study in Riyadh to determine the variables related to nurses’ intention to leave their hospital. Data were collected from three hospitals in Riyadh. Of the 488 respondents, 275 (56.4%) intended to leave their job. Al-Ahmadi [47] collected data from 434 nurses working in nine psychiatric hospitals randomly selected from various geographic regions of Saudi Arabia. Results showed that 37% of nurses had the intention to leave the institution. Most recently, Zaghloul, et al. [48] studied the intention of 276 nurses to stay at a university hospital in Al-Khobar, Saudi Arabia. Findings revealed that about 17% of the sample (47 nurses) agreed that they had intentions to leave. Additionally, more than half of the respondents were not sure exactly whether they intended to leave or not. Studies on health professionals, other than nurses, in Saudi Arabia reported similar findings as well. For example, Al-Ahmadi’s [50] study found that approximately 38% of respondents reported an intention to leave their current hospital. However, the present study is the first to address the issue of turnover intention in the PHC sector in Saudi Arabia.

### Demographic variables and turnover intention

Findings of this study revealed significant associations between turnover intention and demographic variables of gender, age, marital status, dependent children, education level, nursing tenure, organisational tenure, positional tenure, and payment per month. Younger nurses were more likely to indicate turnover intention compared to older nurses, a finding consistent with prior research [51]. On the other hand, older nurses were reported in several studies to be more satisfied with their work and, in turn, less likely to plan on leaving [44,52-56]. Older nurses may have strong personal ties to the organisation and leaving the organisation (before retainment) would be costly and unworthy for them [57-59].

Male respondents had a higher intention to leave their current employment. The literature is not consistent in terms of the relationship between gender and each of employees’ satisfaction and turnover intention. A number of nursing studies support the notion that the female nurses are more satisfied in their work and are more likely to stay [60-64]. Other studies found no relationship between gender and employees’ satisfaction and their intention to leave [44,47,65]. It can be argued that male nurses were less satisfied with their work life and were more intent on leaving their current employment for two reasons: first, male nurses in this study comprised 32.7% (*n* = 166/508) and about 99% of them (*n* = 164) were Saudis. Saudi males are responsible for their families, parents and relatives; thus, they prefer to work in or close to their communities so they can meet their responsibilities. However, contrary to the Saudi female nurses, the Saudi male nurses do not have the opportunity to work in their living areas − Saudi female nurses are given this priority [15]. Another possible cause for gender difference in terms of turnover intention is the poor public image of nursing in Saudi Arabia. Although 36.4% of the nursing workforce in Saudi Arabia are males, community members do not regard nursing as highly as other health disciplines. Male nurses are particularly not well regarded by the community. ‘Hidden turnover’ among Saudi male nurses is a challenge for public healthcare organisations where male nurses work in management or other non-nursing or non-clinical departments and yet are officially counted in the nursing workforce. This kind of ‘turnover’ appears in the public health facilities due to lack of accountability for employee and management departments. In this case, organisational turnover and professional turnover are associated.

Nurses who have never married were more likely to indicate turnover intention. This finding is consistent with the literature [66]. A possible explanation for this finding is that the nurses who have never married were younger compared to the other groups so they may not have the required clinical and life skills to cope with their working environment when it differed from their expectation. Additionally, nurses who have never married may have less family responsibilities so they do not have to consider moving family members when transferring to another organisation [67].

Respondents with children were less likely to indicate an intention to leave than those who had no children. This could be attributed to the responsibilities of parent-nurses ‘as breadwinners’ towards their family members, including children. According to Phillipson and Smit [68], financial commitments to children such as pressure to fund them through university may increase the likelihood for people to remain in their workplace. This was supported in previous research [69,70].

Respondents with an Associate Degree were more likely to indicate turnover intention compared to other groups. While a number of studies found higher status of education to be more related to nurses’ turnover intention [49,64,71-73], others revealed the opposite [4,44]. Moreover, a number of studies found no significant relationship between education status of nurses and their turnover or turnover intention [47,74,75].

In the current study, it could be argued that Associate Degree holders were intending to leave in order to pursue their studies. While they usually study for about three and a half years to graduate with an Associate Degree in nursing, in practice they perform the same tasks as Diploma or Institute graduates. However, when they decide to pursue further study inside the country, they receive no recognition of prior learning and thus must start their studies from scratch [76]. All these factors may negatively influence their commitment to their organisations and profession and push them to leave.

Negative relationships were revealed between turnover intention and years of experience in nursing, with the organisation and in the current position. Turnover intention decreased as the years of experience increased. This finding is consistent with prior research [61,64,77]. It could be argued that nurses with longer years in their jobs may have become used to their work, duties, co-workers, general working environment and the organisation’s system; as a result, they have developed a high level of commitment to their work, position and organisation. Thus, they do not intend to leave their organisation.

Level of salary was significantly associated with the scores of turnover intention. Nurses with lower salary demonstrated higher intent of turnover than higher salary employees. This indicates that salary is important for the satisfaction and retention of the PHC nurses. According to Gardulf et al. [43], salary alone may not be the only factor encouraging nurses to leave, as not being offered an opportunity to discuss the salary and related criteria may also be a contributing factor. For example, nurses may not know why they receive the salary that they do and what to do to improve it.

### Relationship of QWL and turnover intention

The findings indicated that the QWL dimensions explained 19% of the variance in turnover intention. However, the model as a whole (demographics and QWL dimensions) explained 32.1% of variance in nurse’s turnover intention. These findings are similar to the models tested by Tourangeau and Cranley [64], Shader, et al. [54], Gregory, Way, LeFort, Barrett and Parfre [78], Sourdif [73] and Larrabee, et al. [75], which explained 34%, 31%, 31%, 26% and 25.5% of variance in turnover intention.

The work context dimension makes the strongest unique contribution to explaining turnover intention, followed by the work design dimension, β = −.387 and β = −.112, respectively. The ‘work context’ dimension includes a number of variables: management and supervision, co-workers, professional opportunities and work environment. These variables were found in prior research to be associated with turnover intention [54,79].

Evidence was found to support the impact of the ‘work design’ variables on the PHC nurses’ turnover intention. These variables include job satisfaction level, workload, lack of workforce, lack of autonomy, non-nursing tasks, interruptions, limited time to do jobs and patient care [54,80,81].

Although the bivariate correlations between turnover intention and each of the work life/home life and work world dimensions were statistically different from zero, they were not found to be statistically significant contributors to turnover intention among the PHC nurses using multiple regression analysis. However, the impact of these two dimensions on the nurse’s turnover intention cannot be omitted. Factors such as family needs, working hours, salary and public image of nursing were reported in prior research as important predictors of the nurses’ turnover intention [44,46,79,82].

It could be argued that the questionnaire items regarding home life/work life and work world did not cover all the variables of these dimensions. Including additional variables to the scale may explain more variance in nurses intention to leave or stay [64]. For example, a model tested by Boyle, Bott, Hansen, Woods and Taunton [83] explained 52% of variance in ICU nurse intention to stay, because they used a large number of independent variables. As argued by Tourangeau and Cranley [64], the regression model of the current study explained about 32% of variance in nurses’ turnover intention, meaning that approximately 68% of variance remained unexplained. This indicates that there are other important predictor variables to turnover intention of PHC nurses not captured by the model [64].

### Limitations

This study has a number of limitations. First, it used a convenience sampling method that may limit generalization of the findings to PHC nurses in Saudi Arabia. However, it is noted that all of the PHC centres in the Jazan region were included in the study. In addition, the study used a self-reporting survey to collect the data, leaving the interpretation to the participants. Finally, the questionnaires were distributed to the participants through the managers of the PHC centres. These managers may have pressured the nurses to complete the survey in a particular way. However, no such case reports were received by the researchers. Despite these limitations, the study has provided important findings and contributed significantly to the body of research knowledge regarding QWL and turnover intention of PHC nurses. The findings could assist Nurse Managers and policy makers to understand the impact of work life experience on the retention challenges of PHC health professionals, particularly nurses.

### Suggestions for further research

Using a cross-sectional survey design limits the observation of change over time. Further longitudinal study is required to look at changes in QWL and turnover intention of PHC nurses at various points in time. Ideally, participants would be linked at different time points. Such a methodology would enable actual attrition to be monitored against turnover intention.

It would be valuable to conduct an intervention study to improve QWL and retention of PHC nurses considering the findings of the present study. For example, a PHC centre or a small group of PHC centres could be chosen for a longitudinal intervention study to assess the impact of providing supportive facilities for nurses, clearly identifying roles and tasks, offering better payment and professional opportunities on the improving the QWL and retention of nurses. This would help in assessing the impact of such strategies on reducing turnover of PHC nurses.

There is a need to conduct a series of comparative studies focusing on QWL and turnover intention of nurses. These studies may compare nurses to other health professionals in PHC centres and PHC nurses to hospital nurses as well as nurses in public sector to nurses working in private health organisations. Variety in health systems and working environments may produce different determinants of QWL and turnover intention of nursing personnel.

## Conclusions

Nurse turnover is a major challenge for many healthcare services and it interacts with the employees’ QWL. The PHC nurses in this study indicated low satisfaction with their QWL and a high turnover intention. There is a significant association between QWL and turnover intention of PHC nurses. This information could be used to develop appropriate strategies to improve QWL and to reduce the turnover of PHC nurses. Sustaining a healthy work life for PHC nurses is crucial to improve their QWL, increase retention, enhance performance and productivity and promote safe nursing care.

## Competing interests

The authors declare that they have no competing interests.

## Authors' contributions

All of the authors have contributed significantly in the development of this work. M. J. Almalki contributed to the design, collected and synthesized the data used in this paper and wrote the main text. G. FitzGerald and M. Clark contributed to the design, editing and revisions of the manuscript. All authors read and approved the final manuscript.

## Pre-publication history

The pre-publication history for this paper can be accessed here:

http://www.biomedcentral.com/1472-6963/12/314/prepub
